# A Medical Department in the Chicago Medical Library

**Published:** 1884-11

**Authors:** Edmund Andrews, F. C. Hotz, David Graham

**Affiliations:** Com. on Library Chi. Med. Soc.; Com. on Library Chi. Med. Soc.; Com. on Library Chi. Med. Soc.


A MEDICAL DEPARTMENT IN THE CHICAGO PUBLIC LIBRARY.

The Chicago Medical Society wishes to inform the profession that, through its efforts and those of physicians of Cook county, there are 1,200 bound volumes of medical books in the Chicago Public Library ready to be consulted by the profession.

The majority of these works have been recently purchased, and are, in fact, a carefully chosen collection of the best known *recent* medical authorities in English, French and German languages.

This is an initiatory movement toward founding a large medical library in the Northwest, a project in which the whole profession is assumed to take an active interest, and toward which subscriptions of books and money will be collected annually. Moderate yearly additions will soon build up a library in Chicago second only to that of the Surgeon General.

The first year's purchase has included most of standard works on the leading specialties (including dental surgery) and also some works on pharmacy. All these works have been donated outright to the library authorities and will be perpetually cared for and kept on reference by them.

The funds for the purchase consisted of an appropriation of $500 from the society treasury, supplemented by voluntary contributions from the physicians, dentists and druggists of Cook county.

Valuable gifts of old works were also received from some practitioners and deceased physicians' families.

Physicians will find this collection located in the southeast corner of the south room on the upper floor of the library building, where a large table and chairs have been provided for their convenience.

By agreement with Mr. Poole, the books will not be loaned out, but kept in the reference list for consultation, so as always to be found when required. The librarian of the upper floor will show the way to those desiring to consult the books.

There also will be found a good representation of foreign and American medical journals, which will be found catalogued at the librarians counter.

A large and valuable collection of old journals is not yet on the shelves, for lack of money to bind them. Most of these were presented by Dr. Vincent L. Hurlbut.

EDMUND ANDREWS, M. D.,
F. C. HOTZ, M. D.,
DAVID GRAHAM, M. D.,
*Com. on Library Chi. Med. Soc.*

